# Correlation of Bromodomain Protein BRD4 Expression With Epithelial–Mesenchymal Transition and Disease Severity in Chronic Rhinosinusitis With Nasal Polyps

**DOI:** 10.3389/fmed.2020.00413

**Published:** 2020-08-14

**Authors:** Xuanchen Zhou, Zhaoyang Cui, Yiqing Liu, Zhiyong Yue, Fengyang Xie, Ling Ding, Shuai Xu, Jie Han, Hong Zhang

**Affiliations:** ^1^Department of Otorhinolaryngology Head and Neck Surgery, Shandong Provincial Hospital Affiliated to Shandong First Medical University, Jinan, China; ^2^Department of Otorhinolaryngology Head and Neck Surgery, Shandong Provincial Hospital Affiliated to Shandong University, Jinan, China; ^3^Health Management Center, Shandong Provincial Hospital Affiliated to Shandong First Medical University, Jinan, China

**Keywords:** BRD4, chronic rhinosinusitis, nasal polyps, epithelial–mesenchymal transition, disease severity

## Abstract

**Objectives:** This study aimed to explore the relationship between bromodomain-containing protein 4 (BRD4), epithelial–mesenchymal transition (EMT), and disease severity in chronic rhinosinusitis with nasal polyps (CRSwNP).

**Methods:** We performed immunofluorescent (IF) staining to evaluate the expression of BRD4 in the polyp tissues of CRSwNP and inferior turbinate mucosa of healthy controls. The relationship between BRD4 and EMT was evaluated by the BRD inhibitor JQ1 and BRD4 siRNA in primary human nasal polyp–derived epithelial cells. Disease severity was scored by using the Lund–Mackay scores of paranasal sinus computed tomography (CT) scans.

**Results:** The expression of BRD4 in patients with CRSwNP was significantly higher than that in healthy controls. The loss of BRD4 function by the BRD inhibitor JQ1 and BRD4 siRNA resulted in the reduction of E-cadherin, increasing vimentin, and Snai1 mRNA expression. Moreover, the expression of BRD4 was related to the total CT scan scores (*r* = 0.4682, *P* = 0.0210).

**Conclusions:** BRD4 had higher expression in CRSwNP than in healthy controls and might be associated with EMT in CRSwNP. BRD4 mRNA expression was associated with disease severity in CRSwNP.

## Introduction

Chronic rhinosinusitis (CRS) is highly prevalent, influencing ~ 11–15% of the adult people (1, 2), and contributing to direct health care costs of $11 billion each year in the European area (3). Chronic rhinosinusitis refers to a heterogeneous group of diseases with common symptoms and clinical findings, but different pathophysiologies. It has been divided into CRS with nasal polyps (CRSwNP) and CRS without nasal polyps basing on whether nasal polyps are present or absent (4). CRSwNP is a chronic inflammatory disease characterized by the inflammation of the nasal mucosa, nasal obstruction, and the growth of nasal polyps (NP) (3). Nasal polyps are infiltrated with multiple inflammatory cells, such as eosinophils, neutrophils, T-helper type 2 (T_H_2) cells, macrophages lymphocytes, innate lymphoid cells, and mast cells (5, 6). The pathogenesis of CRSwNP that is related to T_H_2-based inflammation has been shown in some published literatures (7–9). The evidence shown above demonstrates that the inflammation mainly contributes to the pathogenic process of CRSwNP.

Tissue remodeling is considered a typical response to chronic inflammation (10, 11). It is a dynamic process that involves the structural rearrangement of tissues. Tissue remodeling is also concerned with chronic inflammation of the upper airway, such as allergic rhinitis or CRS, including inflammatory cell infiltration, basement membrane thickening, subepithelial edema, and fibrosis (11, 12).

Epithelial–mesenchymal transition (EMT) is considered to be a core process of tissue remodeling. It has been reported to play an important role in embryonic development wound healing, fibrosis, tumor invasion, and metastasis (13). The main biological role of EMT is to repair tissue damage caused by trauma and inflammation by inducing epithelial cells to produce fibroblasts. Cells undergo a series of changes during EMT, such as the loss of cell polarity, loss of epithelial markers, loss of tight intercellular connections and adhesions, and the rearrangement of cytoskeletons (14). E-cadherin and vimentin, which represent epithelial and interstitial characteristics, respectively, are important proteins in the EMT process. Decreased expression of E-cadherin, increased vimentin, and morphogenetic mesenchymal cell–like transformation are important manifestations of EMT (15). Additionally, Snai1, Slug, Twist, ZEB, and so on, are increased during EMT to inhibit the expression of E-cadherin (16).

A previous study has reported that EMT played a role in the pathogenesis of CRSwNPs (11). The expressions of E-cadherin, N-cadherin, vimentin, fibronectin 1, Snai1, Slug, and Twist in NPs and inferior turbinates from CRSwNP were significantly higher than in inferior turbinates from healthy subjects (17). Studies have shown that transforming growth factor β1 (TGF-β1) promoted EMT in bronchial epithelial cells and primary nasal epithelial cells (18, 19). However, the molecular mechanism of EMT is poorly studied in CRSwNP.

Bromodomain-containing protein 4 (BRD4), as a member of the brominated structure and ultraterminal structure family, plays various physiological and pathological roles by binding acetylated histones with brominated domain throughout the cell cycle (20). Most of the research on BRD4 focuses on promoting the occurrence and development of tumors and mainly involves the occurrence of EMT in tumors. Studies have shown that BRD4 is involved in EMT and airway remodeling in asthma, airway inflammation, and pulmonary fibrosis (21–23). However, it has not been reported if BRD4 is involved in the occurrence of EMT in CRSwNP.

In this study, we evaluated the expression of BRD4 in human NP tissues of CRSwNP and healthy controls at the mRNA and tissue levels and analyzed the correlation between BRD4 expression and CRSwNP disease severity. The role of BRD4 on EMT in CRSwNP was assessed using a BRD4-specific siRNA knockdown or BRD inhibitor (JQ1) in primary human NP–derived epithelial cells (hNPDECs).

## Materials and Methods

### Study Subjects

Adult patients with CRSwNP (*n* = 24) and a healthy control group (*n* = 24) were recruited from Shandong Provincial Hospital affiliated to Shandong First Medical University, China. All the patients gave written consent to undergo tissue collection at the time of surgery. Nasal polyp tissue was harvested at the time of endonasal sinus surgery from 24 patients with CRSwNP [median age 43 years (range, 19–65 years)]. Patients undergoing septal or turbinate surgery for functional reasons were enrolled as healthy control subjects (*n* = 24) and the inferior turbinate was sampled during surgery. Diagnosis was confirmed, according to the European Position Paper on Rhinosinusitis 2012 criteria (3). Unilateral NP disease, cystic fibrosis, primary ciliary dyskinesia, nasal malignancies, and fungal rhinosinusitis formed the exclusion criteria. The patient's characteristics are listed in Table 1. Asthma was clinically diagnosed according to the Global Initiative for Asthma guidelines, based on a proof of reversibility of forced expiratory volume in 1 s ≥ 12% after the inhalation of salbutamol. Aspirin-induced asthma syndrome was clinically confirmed asthma with a history of aspirin intolerance. The disease severity was evaluated by scoring computed tomography (CT) scans before surgery. All subjects gave written informed consent. The study was approved by the ethics committee of the Shandong Provincial Hospitals affiliated to Shandong First Medical University.

**Table 1:** Patient characteristics∇.

	**CRSwNP (*n =* 24)**	**Controls (*n =* 24)**
Age (years)	43 (19–65)	31 (20.5–53)
Gender (male/female)	12/12	10/14
Asthma (yes/no)	2/21	1/23
AIA (yes/no)	1/23	0/24
Atopy (yes/no)	7/17	6/18
Nasal steroids (yes/no)	0/24	2/22

*∇data are given as median range. AIA, aspirin intolerant asthma*.

### Immunofluorescence

All the NP tissues and control inferior turbinate specimens were washed with phosphate-buffered saline and then embedded in paraffin. Tissue wax blocks were sectioned with a Leica microtome (Leica, Wetzlar, Germany) to obtain a thickness of 4 mm. Rabbit polyclonal anti-BRD4 antibody (ab128874, 1:100 diluted) was from Abcam. The expression of BRD4 on paraffin sections of nasal biopsies was then studied by immunofluorescent (IF) staining. The paraffin sections were imaged with a confocal microscope (LSM 700; Zeiss). All these operations were adapted from this previous study (24).

### Stimulation of EMT by Cultures of Primary hNPDECs and Primary Human Nasal Epithelial Cells

For the investigation of BRD4, E-cadherin, vimentin, and Snai1 mRNA expression from primary hNPDECs and primary human nasal epithelial cells (hNECs) cultures, fresh endonasal tissues were utilized. Primary hNPDECs were collected by gently scraping the epithelial surface of the NP tissues with a convex surgical blade and cultured as described in a previous study (25). In brief, disassociated epithelial cells were first transferred to the 50-mL conical tube and then centrifuged at 200 *g* for 5 min and resuspended in the bronchial epithelial cell growth medium (Lonza, Basel, Switzerland). Lastly, these primary cells were plated with type IV collagen–coated six-well tissue culture plates and incubated at 37°C and 10% CO_2_. The culture medium was replaced every 3 days. Primary hNECs were isolated from inferior turbinates of healthy controls and were cultured (26). For EMT induction, recombinant human TGF-β1 (ReliaTech GmbH, Wolfenbüttel, Germany) was added to the primary cells and preformed as noted in a previous study (17). The total RNA extraction was performed thereafter.

### Real-Time Quantitative Polymerase Chain Reaction

Tissue homogenates were prepared from the polyp tissue of patients with CRSwNP and from the inferior turbinates of controls. Primary hNPDECs and hNECs were collected after culture. The total RNA was isolated according to the manufacturers' protocol (RNeasy mini kit; Qiagen, Germantown, MD, USA). cDNA was synthesized using Superscript II RT (Invitrogen, Carlsbad, CA, USA) and oligo (dT). A set of primer sand probes was designed and optimized for each of the genes BRD4, E-cadherin, vimentin, Snai1, and the housekeeping gene GAPDH. The primer pairs used in the study are shown in Table 2. All polymerase chain reactions (PCRs) were performed in triplicate. Relative gene expression was normalized to the housekeeping gene GAPDH as a reference and was calculated using the comparative 2-ΔΔCt method.

**Table 2:** The sequences of PCR primers used in this study.

**Gene**	**Forward primer**	**Reverse primer**
BRD4	5′-CCCTGAAGCCGTCCACACT-3′	5′-TTCTCAGCTTGAGGTTTCCTTTTC−3
E-cadherin	5′-AGTGCCAACTGGACCATTCA-3′	5′-TCTTTGACCACCGCTCTCCT-3′
Vimentin	5′-CATCCTTCTCACTGCCATGGA-3′	5′-AGGCAGAGGACACAGAACCAGA-3′
Snai1	5′-TGCCCTCAAGATGCACATCCGA-3′	5′-GGGACAGGAGAAGGGCTTCTC-3′
GAPDH	5′-TGCACCACCAACTGCTTAGC-3′	5′-GGCATGGACTGTGGTCATGAG-3′

### Gene Silencing of BRD4 in Primary hNPDECs

Transfection of hNPDECs was performed using BRD4 siRNA (siBRD4) and negative control siRNA (siCONT) obtained from Ambion (Thermo Fisher Scientific) (27). The cells were transfected with 50 nM siCONT or 50 nM siBRD4 and 0.3% Lipofectamine 3000 (Life Technologies), incubated for 20 h, and removed, and fresh medium was added to the wells. After 66 h (total culture time), the cells were treated with or without 10 ng/mL TGF-β1 and were incubated at 37°C for an additional 20 h. Cells were collected and stored at −80°C until assayed for mRNA expression by quantitative reverse transcriptase (qRT)–PCR as described above. Experiments were performed on primary hNPDECs isolated from seven patients.

### Treatment of JQ1 in Primary hNPDECs

Isolated primary hNPDECs were also treated with 0.5 μM JQ1 in the presence or absence of 10 ng/mL TGF-β1, following a total culture time of 60 h, and the cells were incubated at 37°C for an additional 20 h. Cells were collected and stored at −80°C until assayed for mRNA expression by qRT-PCR as detailed above. Media was collected and stored at −80°C until assayed for EMT markers. Experiments were performed on primary hNPDECs isolated from seven patients.

### Radiological Evaluation of Disease Severity

The Lund–Mackay score was used to radiologically score sinus opacity on paranasal sinus CT scans, which enabled the assessment of disease severity and NP size. The Lund–Mackay score is a score system adopted to quantify the severity of CRS in rhinology (28).

### Statistical Analysis

Standardized data were collected, unless otherwise noted. All statistical analyses were conducted using GraphPad Prism (GraphPad Software). Two sample comparisons were analyzed to assess the statistical significance using paired/unpaired Student *t*-test. For all other comparisons, a repeated-measures one-way analysis of variance (ANOVA) was adopted to analyze the data. *P* < 0.05 was considered statistically significant. Data were expressed as mean ± SD.

## Results

### BRD4 Expression Levels and Distribution in NP Tissues and Controls

**Figure 1:**
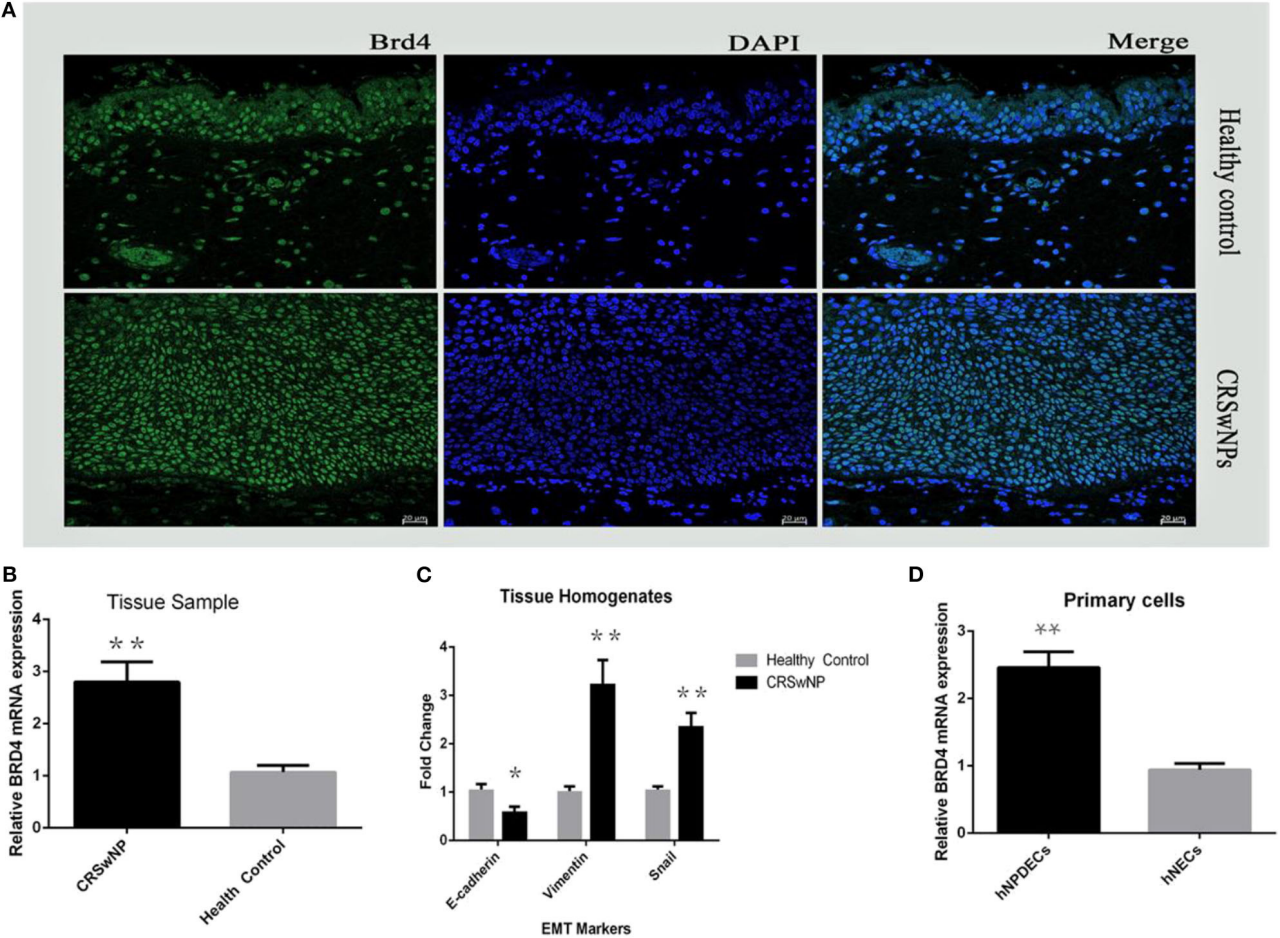
**(A)** Immunofluorescence staining (DAPI) on 20-μm slides from fresh frozen NP tissue (CRSwNPs) and control inferior turbinate mucosa (healthy control) performed with polyclonal antibodies against rabbit BRD4. **(B)** The relative mRNA expression of BRD4 in tissue homogenates from patients with CRSwNP (*n* = 24) and controls (*n* = 24) after qRT-PCR analysis normalized to the housekeeping gene GAPDH. **(C)** Plot of EMT marker E-cadherin, vimentin, and Snai1 in CRSwNPs and healthy control. **(D)** The relative mRNA expression of BRD4 after qRT-PCR analysis in primary hNPDECs and hNECs isolated and cultured from NP tissues (*n* = 7) and healthy nasal inferior turbinate tissues (*n* = 7). Data were expressed as mean ± SD. The Student test was used for statistical comparison. *P < 0.05, **P < 0.01 vs. controls.

First, we assessed the expression and cellular location of BRD4 in the nasal mucosa. As shown in Figure 1A, BRD4 was localized in the cell nucleus of both NP epithelial cells from CRSwNP and nasal epithelial of normal controls in the representative IF images. Much less staining was present in the healthy controls. Next, in order to evaluate whether BRD4 was altered in NP tissues from patients with CRSwNP, we determined the mRNA expression of BRD4. As shown in Figure 1B, BRD4 mRNA levels were significantly higher in polyp tissues from patients with CRSwNP than those in inferior turbinate tissues from control subjects (*P* < 0.05). Thereafter, we investigated the mRNA expression of EMT markers using qRT-PCR in tissue samples from CRSwNP and controls. The expression of vimentin and Snai1 in NPs was significantly higher (*P* < 0.01) than that in inferior turbinates from healthy objects (Figure 1C), and the expression of E-cadherin in CRSwNP was significantly lower than that in healthy controls (^**^P < 0.01).

### BRD4 mRNA Expression in Primary hNPDECs and hNECs

To confirm the expression of BRD4 on epithelial cells, mRNA was extracted from the cultures of primary hNPDECs and hNECs. Bromodomain-containing protein 4 mRNA was detected in both types of primary cells. As shown in Figure 1D, the mRNA levels of BRD4 were significantly up-regulated in primary hNPDECs compared to primary hNECs. Statistical difference was defined as *P* < 0.05 (^*^) or *P* < 0.01 (^**^).

Then, we unsuccessfully tried to quantify BRD4 protein expression by Western blotting using several different commercially available antibodies in both nasal tissues and primary epithelial cells, but failed. These findings above proved that the expression of BRD4 mRNA was significantly up-regulated in the NP epithelial cells of patients with CRSwNP, but not in the control group.

### BRD4 Inhibition Changes the Expression of EMT Markers in Primary hNPDECs

**Figure 2:**
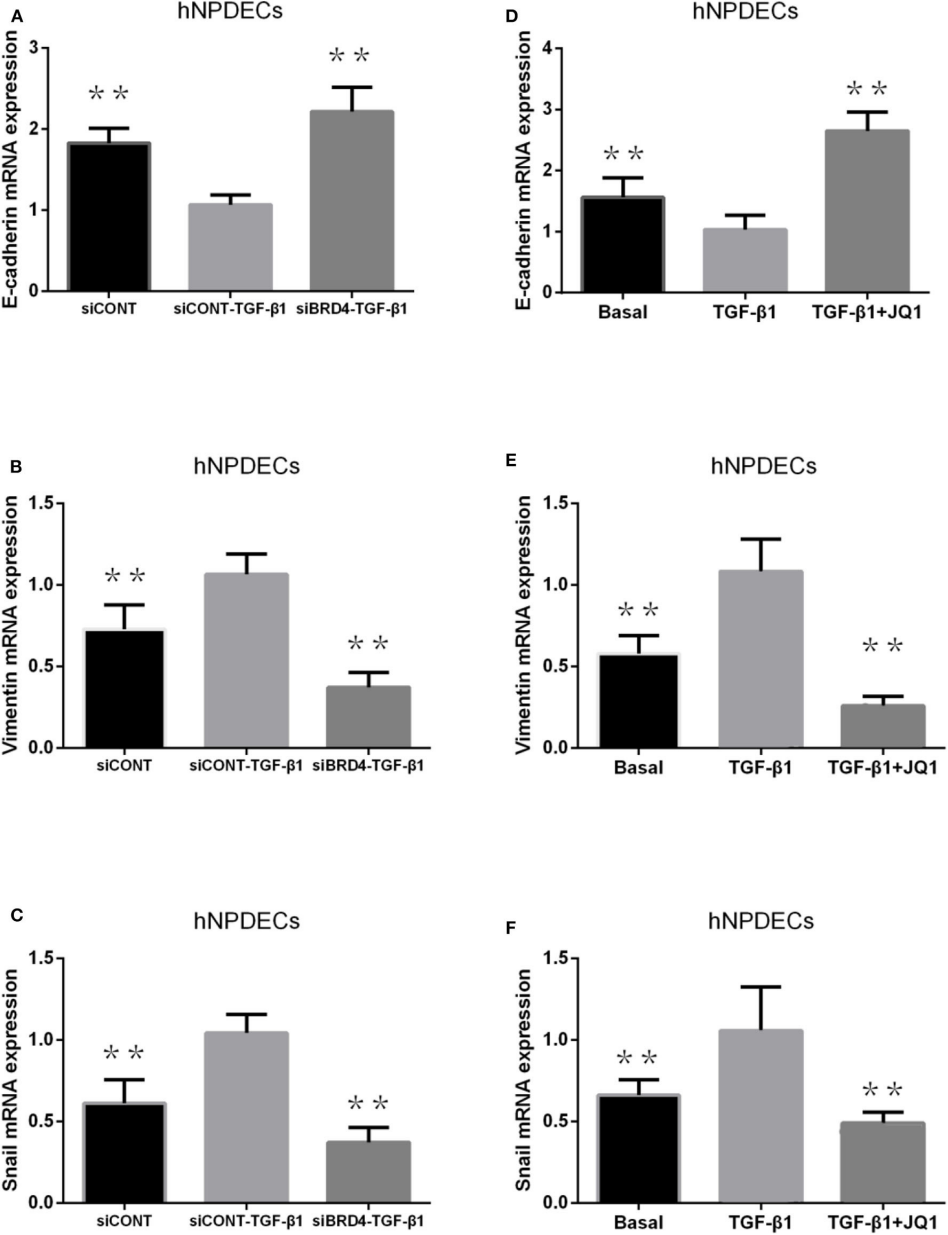
Effect of BRD4 silencing on TGF-β1–induced EMT marker production (E-cadherin, vimentin, and Snai1) production in human primary hNPDECs **(A–C)**. Human primary hNPDECs were either transfected with siBRD4 or siCONT. Effect of BRD4 inhibitor JQ1 on TGF-β1 induction EMT marker production in human primary hNPDECs **(D–F)**. E-cadherin, vimentin, and Snai1 mRNA expression was assessed by qRT-PCR. Fold change was calculated relative to siCONT–TGF-β1. All data are displayed as mean ± SD. The one-way ANOVA was used for statistical comparison. *P < 0.05, **P < 0.01 vs. controls.

Transforming growth factor β1 has been proved to be able to simulate the process of EMT in primary bronchial cells and nasal epithelial cells. We used two methods to analyze the effect of BRD4 on EMT in primary hNPDECs: (1) BRD4-specific siRNA knockdown and (2) treatment with the chemical inhibitor JQ1. We performed BRD4 siRNA knockdown in primary hNPDECs and confirmed BRD4 silencing at the mRNA (71% decrease). Absence of E-cadherin and the acquisition of vimentin are two critical steps in EMT. In hNPDECs transfected with siBRD4, there was a significant increase in TGF-β1–induced E-cadherin mRNA expression (^**^P < 0.01, Figure 2A) and significantly decreased expression of vimentin and Snai1 (^**^P < 0.01, Figures 2B,C). In contrast, in hNPDECs treated with JQ1, the mRNA expression of E-cadherin, vimentin, and Snai1 presented a similar variation tendency (^**^P < 0.01, Figures 2D–F) as that shown by siBRD4.

### Correlation of BRD4 mRNA and EMT Markers mRNA Expression With CT Scan Scores

**Figure 3:**
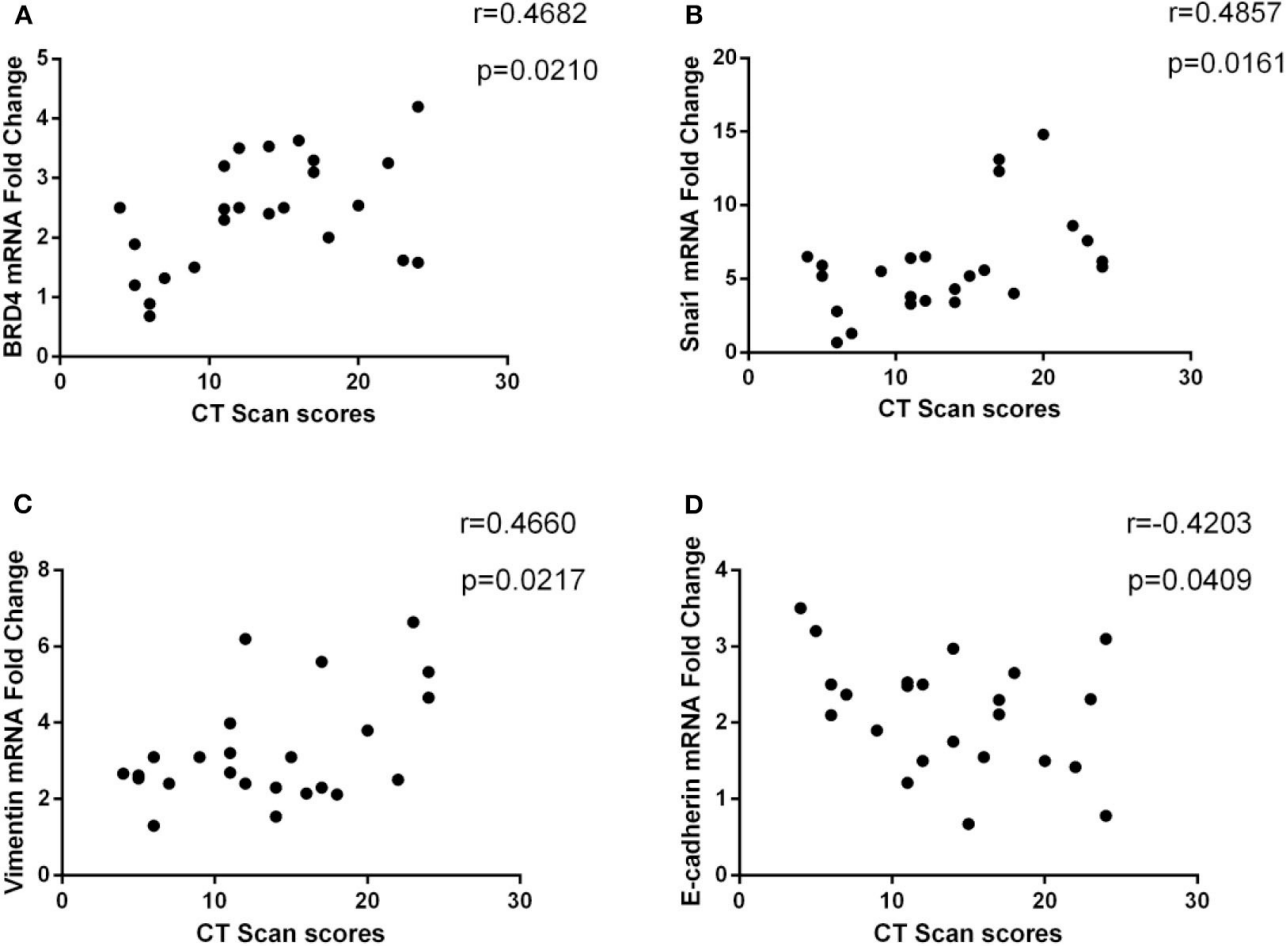
**(A)** Correlation between CT scan score (Lund–Mackay) and BRD4 mRNA expression in CRSwNP (*n* = 24). **(B–D)** The association between CT scan score and E-cadherin, vimentin, and Snai1 mRNA expression in CRSwNP (*n* = 24) was presented. The Spearman rank correlation coefficient was used for statistical comparison.

We investigated the relationship between BRD4 and disease severity in patients with CRSwNP. As shown in Figure 3A, the mRNA expression of BRD4 positively correlated with the CT scan scores (*r* = 0.4682, *P* = 0.021). We also studied the correlation of EMT biomarker mRNA expression with disease severity in patients with CRSwNP and found that E-cadherin, vimentin, and Snai1 were significantly associated with CT scan scores (Figures 3B–D). There are statistically significant differences among all the results (*P* < 0.05).

## Discussion

Chronic rhinosinusitis with nasal polyps is characterized by chronic inflammation of the nasal mucosa, nasal obstruction, and growth of NPs, which brings a serious psychological burden and economic pressure upon the patient. Tissue remodeling is a typical pathological change in CRSwNP, and EMT is a key process involved in tissue remodeling. Recently, data have supported the role of EMT in CRSwNP (17, 29). Studies have shown that BRD4 participates in tissue remodeling or EMT in carcinoma, asthma, and pulmonary fibrosis (21, 22, 30). However, the relationship between BRD4 and EMT of CRSwNP remains unclear. In the present study, we showed the expression of BRD4 in NP tissues and healthy controls at the mRNA and the cellular level and revealed that BRD4 was associated with EMT. Bromodomain-containing protein 4 and EMT markers were correlated with disease severity of CRSwNP. Furthermore, treatment of primary hNPDECs with siBRD4 and BRD4 inhibitor JQ1 resulted in a significant change in EMT markers. This indicated that BRD4 might contribute to EMT pathophysiology of CRSwNP.

Bromodomain-containing protein 4 has been found in many other types of cells (31, 32) but has never been reported in the nasal tissues. By immunofluorescence, we are the first to confirm that BRD4 protein was located and expressed in the nucleus of NP epithelial cells (Figure 1A). Bromodomain-containing protein 4 had overexpression in NP tissues compared with the control group, but there was no quantitative analysis because of limited experiment condition. By qRT-PCR, we demonstrated a significantly higher expression level of BRD4 mRNA in NP tissues of CRSwNP than in healthy nasal inferior turbinate tissue (Figure 1B). The mRNA expression level of BRD4 increased by different degrees in almost all CRSwNP patients. Furthermore, in primary cell cultures, BRD4 mRNA expression also significantly increased in hNPDECs compared to hNECs. E-cadherin, vimentin, and Snai1 are key markers in EMT formation. Our results demonstrated that EMT existed in NP tissues by detecting EMT biomarkers between CRSwNP and controls. E-cadherin, vimentin, and Snai1 are all significantly different in NP tissues from control turbinate tissues, consistent with the results of previous studies (Figure 1C) (17). Bromodomain-containing protein 4 has been demonstrated to be expressed in the nucleus of different types of cells, including oral squamous cell carcinoma, salivary adenoid cystic carcinoma, umbilical vein endothelial cells, and epithelial cell of the trachea. Our finding will further enrich the library of BRD4 expression in human body. Meanwhile, the EMT will provide us a new sight to explore the pathogenesis of CRSwNP.

Epithelial–mesenchymal transition is a key process in tissue remodeling in CRSwNP, but researches have seldom focused on it. We demonstrated, for the first time, that BRD4 is involved in the pathophysiology of EMT in CRSwNP. Our results show that BRD4 up-regulation was related to NP epithelial cellular EMT. In EMT, vimentin and Snai1 expressions increase, but E-cadherin expression decreases. We found that BRD4 silencing led to a significant reduction in vimentin and Snai1 production and a significant increase in E-cadherin production in TGF-β1–induced primary hNPDECs (Figures 2A–C). Blocking of BRD4 with JQ1 also showed similar results as silencing BRD4 (Figures 2D–F). Thus, we propose that decreased BRD4 expression might change the expression of EMT biomarkers and attenuate EMT formation in hNPDECs. However, further studies needed to determine how BRD4 modulates EMT in CRSwNP. Bromodomain-containing protein 4 played an important role in asthma inflammation and remodeling. Tian et al. have reported that nuclear factor κB (NF-κB)–inducible BRD4 activity could mediate the mice asthma inflammation and remodeling induced by cat dander. Another study shows that NF-κB/RelA-BRD4 pathway played a complex role in allergic asthma inflammation. Combining with the role of BRD4 in the EMT of CRSwNP, BRD4 is involved in the inflammation and remodeling of the entire upper airway. Inhibition of BRD4 might have the potential to control the inflammation of nasal cavity, trachea, and lung.

In this study, we found that the mRNA expressions of BRD4 and EMT markers were related to the clinical data, such as nasal CT scores (Figure 3). Bromodomain-containing protein 4, vimentin, and Snai1 positively correlated with CT scores, and E-cadherin was negatively related. These data suggest that the expression of BRD4 and EMT markers might be useful indicators of disease severity in CRSwNP patients, but this needs to be confirmed by further studies in a larger study population. We do not know if this is a diagnostic marker and accurate judgment, because CRSs without NPs have not been studied. In our study, Spearman rank correlation method was used to evaluate the correlation of the expression of BRD4 and disease severity. As we know that the Spearman rank correlation method is widely used in different areas to evaluate the correlation between two variables. In this study, our study only demonstrated that BRD4 or EMT marker was associated with disease severity by using Spearman rank correlation, in fact, not a very exact event. In a previously published report, the authors also utilized this method to access PD-1 mRNA expression, TGF-β, IL-5, or IL-10 in NP tissue correlation with total CT scan scores (33). Therefore, we believe that Spearman rank correlation is a reliable method. There is a controversy that people should not represent the severity of inflammation with CT scores instead of inflammatory markers in secretion or tissue. However, although considering CT scores as the severity of inflammation is debatable, CT scores is an easy, simple, and cheap test method for the patients. Furthermore, a previous study with similar results has been recognized by scholars (33). Therefore, it is worth popularizing in the clinical practice.

The limitation of this study was that we could not quantify BRD4 protein expression in NP tissues of patients with CRSwNP. Our attempts to quantify BRD4 expression using commercial antibodies for many times were still unsuccessful. A published report also shows a similar phenomenon (27). Then, we utilized other substitute reagents to repeat this Western blot test, and the result still failed. Although we determined that BRD4 was associated with EMT in CRSwNP, the specific regulatory mechanisms and molecular pathways remain unclear. Further research is needed to determine that in the future. The expression of BRD4 and EMT markers associated with disease severity may have an error because of the small study population in this study.

In conclusion, the novel findings of this study suggest that increased BRD4 expression may play an important role in the pathogenesis of EMT in CRSwNP. The correlation of the expression of BRD4 and EMT markers with radiologic evaluation highlights the importance of their role in the pathogenesis of CRSwNP.

## Data Availability Statement

The raw data supporting the conclusions of this article will be made available by the authors, without undue reservation, to any qualified researcher.

## Ethics Statement

The studies involving human participants were reviewed and approved by Ethics Committee of the Shandong Provincial Hospitals affiliated to Shandong First Medical University. The patients/participants provided their written informed consent to participate in this study.

## Author Contributions

XZ and JH designed research. XZ and HZ performed research. YL and ZY contributed new reagents and analytic tools. ZC and FX analyzed data. XZ, LD, and SX wrote the paper. All authors revised the manuscript and approved the final version. All authors contributed to the article and approved the submitted version.

## Conflict of Interest

The authors declare that the research was conducted in the absence of any commercial or financial relationships that could be construed as a potential conflict of interest.

## Abbreviations

BRD4: bromodomain-containing protein 4
CRSwNP: chronic rhinosinusitis with nasal polyps
NP: nasal polyps
EMT: epithelial–mesenchymal transition
hNPDECs: human nasal polyp–derived epithelial cells
hNECs: human nasal epithelial cells
IF: immunofluorescent
GINA: Global Initiative for Asthma
AIA: aspirin-induced asthma
CT: computed tomography.
