# Corrigendum: Correlation of Bromodomain Protein BRD4 Expression With Epithelial–Mesenchymal Transition and Disease Severity in Chronic Rhinosinusitis With Nasal Polyps

**DOI:** 10.3389/fmed.2020.604072

**Published:** 2020-10-29

**Authors:** Xuanchen Zhou, Zhaoyang Cui, Yiqing Liu, Zhiyong Yue, Fengyang Xie, Ling Ding, Shuai Xu, Jie Han, Hong Zhang

**Affiliations:** ^1^Department of Otorhinolaryngology Head and Neck Surgery, Shandong Provincial Hospital Affiliated to Shandong First Medical University, Jinan, China; ^2^Department of Otorhinolaryngology Head and Neck Surgery, Shandong Provincial Hospital Affiliated to Shandong University, Jinan, China; ^3^Health Management Center, Shandong Provincial Hospital Affiliated to Shandong First Medical University, Jinan, China

**Keywords:** BRD4, chronic rhinosinusitis, nasal polyps, epithelial–mesenchymal transition, disease severity

In the published article, there was an error in **affiliation 3**. Instead of “Minimally Invasive Urology Center, Shandong Provincial Hospital Affiliated to Shandong First Medical University, Jinan, China,” it should be “Health Management Center, Shandong Provincial Hospital Affiliated to Shandong First Medical University, Jinan, China.”

The authors apologize for this error and state that this does not change the scientific conclusions of the article in any way. The original article has been updated.

